# Heart teams in the Netherlands: From teamwork to data‑driven decision-making

**DOI:** 10.1007/s12471-020-01452-8

**Published:** 2020-08-11

**Authors:** E. Wierda, D. van Veghel, A. Hirsch, B. A. J. M. de Mol

**Affiliations:** 1 Department of Cardiology, Dijklander Hospital, location Hoorn, Hoorn, The Netherlands; 2 Department of Cardiology and Cardiothoracic Surgery, Catharina Hospital Eindhoven, Eindhoven, The Netherlands; 3 Department of Cardiology and Radiology and Nuclear Medicine, Erasmus MC, University Medical Center Rotterdam, Rotterdam, The Netherlands; 4 Department of Cardiothoracic Surgery, Amsterdam UMC, University of Amsterdam, Amsterdam, The Netherlands

**Keywords:** Heart team, Teamwork, Cardiovascular intervention, Shared decision-making

## Abstract

For all patients with cardiovascular disease requiring an intervention, this is a major life event. The heart team concept is one of the most exciting and effective team modalities to ensure cost-effective application of invasive cardiovascular care. It optimises patient selection in a complex decision-making process and identifies risk/benefit ratios of different interventions. Informed consent and patient safety should be at the centre of these decisions. To deal with increased load of medical data in the future, artificial intelligence could enable objective and effective interpretation of medical imaging and decision support. This technical support is indispensable to meet current patient and societal demands for informed consent, shared decision-making, outcome improvement and safety. The heart team should be restructured with clear leadership, accountability, and process and outcome measurement of interventions. In this way, the heart team concept in the Netherlands will be ready for the future.

## Dutch contribution to the field

- The SYNTAX trial assessing the optimal revascularisation strategy (coronary-artery bypass grafting versus percutaneous coronary intervention) in patients with three-vessel or left main coronary artery disease, mentioned the heart team concept for the first time and evoked discussion.
- First author of the manuscript, published in the New England Journal of Medicine in 2009, was Professor P.W. Serruys, interventional cardiologist at the Erasmus University Medical Center.
- Outcome measurement of interventions and institutional performance are monitored by the Netherlands Heart Registration (NHR), a joint effort of the specialities cardiology and cardiac surgery.
- Innovative Dutch research is conducted on image recognition, natural language processing and decision support systems to guide heart team decisions.

## Introduction and background

The evolution of cardiovascular care in the last seven decades is fuelled by better understanding of pathophysiology and advances in treatment. Cardiac surgery became the showcase of complex and multidisciplinary interaction of experts, in areas ranging from patient selection to intervention, postoperative care and follow-up. Dutch cardiologists and cardiac surgeons embraced multidisciplinary decision-making from the start. Initially, the heart team was composed of both the referring cardiologist, and the cardiac surgeon and cardiologist in the cardiac surgical centre.

Compared with those early days, the aims of good decision-making remained unchanged but the requirements for risk control have changed significantly. This change has been pushed by advanced imaging techniques and availability of new treatment options, such as minimally invasive surgery and catheter-based interventions. Between 1980 and 2010, heart teams consisting of the interventional cardiologist and cardiac surgeon from the cardiac surgical centre were responsible for intake and preoperative care. The role of the referring cardiologist became diluted. The heart team dealt mostly with decisions as to whether a patient could benefit from valve surgery or from revascularisation by percutaneous coronary intervention or coronary artery bypass grafting. The SYNTAX study mentioned the heart team concept for the first time and evoked discussion [1, 2]. Due to its complexity, heart teams for grown-up congenital heart disease (GUCH) and heart transplantation resembled the multidisciplinary decision-making model common in oncology and included more and different disciplines [3]. Today, after a screening intake by the general heart team, patients are referred to specialty heart teams, such as electrophysiology, catheter-based valve interventions, endocarditis and heart failure [4, 5]. See Tab. 1 for an overview of the different general and specialist heart teams.

**Table 1:** Overview of general and specialist heart teams

Type of heart team	Team members (excluding referring cardiologist and planner)
General	Interventionalist, surgeon, imaging cardiologist
Catheter-based valve interventions	Interventionalist, surgeon, imaging cardiologist/radiologist, anaesthesiologist, intensive care specialist, geriatricians, specialist nurse
Electrophysiology	Electrophysiologist, (device), electrophysiologist (ablation), imaging cardiologist, anaesthesiologist
Endocarditis	Surgeon, imaging cardiologist/radiologist, infectious disease specialist, microbiologist
Heart failure	Heart failure cardiologist, imaging cardiologist, electrophysiologist (device), specialist nurse
GUCH	GUCH cardiologist, interventionalist and surgeon specialised in GUCH, imaging cardiologist/radiologist, electrophysiologist, specialist nurse
Heart transplantation/LVAD	Heart failure specialist, transplantation cardiologist, imaging cardiologist, surgeon, internal medicine specialist, specialist nurse, psychologist

*GUCH* grown-up congenital heart disease, *LVAD* left ventricular assist device

In this contribution we will discuss the advantages and challenges associated with the current practice of the heart team concept. Processing of overload of conventional and novel imaging requires new expertise, resources and time [6, 7]. We make a case that the heart team should improve shared decision-making and outcome measurement of the process. The heart team should not only move towards an intervention but also design a patient-tailored monitoring and treatment pathway for all cardiovascular diseases [8].

## The heart team—definitions and roles

**Fig. 1:**
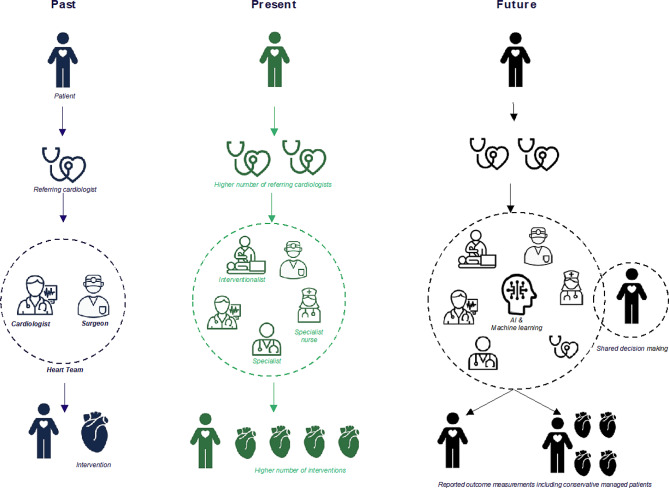
Past, present and future of the heart team concept

In the early days, task distribution between cardiac surgeons and cardiologists was easier to define. The cardiologist was responsible for referral and preoperative and postoperative care. Surgical capacity was limited resulting in waiting lists [9]. The landscape changed with the developments in the management of cardiovascular disease, such as catheter-based interventions for coronary artery disease and valve disease. Cardiac surgeons and interventional cardiologists had to agree on the best indications based on case information, interpretation of guidelines and best practices: not an easy task [10, 11]. With the availability of national and European guidelines and emphasis on the quality of decision-making by the professional societies, the process and structure of a heart team became a formal requirement to justify reimbursement of the intervention [12–14]. See Fig. 1 and Tab. 2 for an overview of the main developments of heart teams in the Netherlands: an increased number of referring cardiologists, the development of interventional cardiology, increased number of team members and cardiovascular interventions.

**Table 2:** Changed variables in heart team decision-making

Patient	– Ageing population with more comorbidity – Higher standards on informed consent – Less paternalistic patient-doctor relationship – Increased consumer mentality with higher demands on information and speed of process of intervention
Referring cardiologist	– Higher number of referring cardiologists – Hospital mergers working at different locations – Increased part-time work complicating communication
Heart team	– Increased complexity of cardiovascular disease with increased morbidity – Innovation and increased number and complexity of non-invasive preoperative imaging techniques – Higher number of team members, involved specialties and paramedics – Digital health records and cardiovascular imaging data with different vendors between hospitals complicating data transfers
Intervention	– Increased number of cardiovascular interventions – Increased complexity of cardiovascular interventions – Hospitals mergers with different locations complicating patient transfer
Professional and societal supervising authorities	– Stricter regulations for patient care in guidelines produced by professional societies – Higher requirements for quality registries – Higher demand for transparency in patient outcomes of cardiovascular interventions – Increased coverage in media on malfunction and adverse events

The advantage of a multidisciplinary heart team is the presence of structure for best available reproducible decision-making while preventing specialty bias for optimal patient care [13]. Potentially professional and institutional interests drive the indication for a surgical or a catheter intervention [15, 16]. Some view the heart team as an institutional illusion to cover conflicts of interests [17]. The critics were served by a recent BBC broadcast on the EXCEL trial and professional differences of opinion between cardiac surgeons and cardiologists about the best treatment of left main coronary artery disease [18].

## Specialty heart team and technological advancements

High-quality heart team decision-making depends on strong logistic and administrative support, which can only be provided in the case of comprehensive and adaptive electronic patient records. Combining data including imaging data from referring hospitals remains troublesome in practice.

Newer imaging techniques, such as high-resolution cardiac CT and 3D echocardiography, contribute to better decision-making but their interpretation requires specific expertise. Strict guidelines for the information required to enter the process and objective interpretation of images based on algorithms and machine learning can prevent wasting time debating the interpretation and the need to assemble all the experts [4–7]. Possibilities include image recognition, natural language processing and decision support systems [19]. Other technological advancements for the heart team include 3D printing of devices or valvular heart disease, wearable patient technology for monitoring and big-data analytics.

Decisions are increasingly complex due to advances in imaging and treatments, available evidence and guidelines, combination of electronic patient records and the logistical presence of the experts needed. These developments make it more complex to plan meetings with team members and arrange communication between heart teams and referring hospitals, but this is also indispensable. Communication should also include newer innovative ways with other participating members of the heart team (e.g. referring cardiologist) and the patient and his or her family.

## Shared decision-making and informed consent

For all patients with cardiovascular disease requiring an intervention, this is a major life event. In the heart team the focus is on technical aspects and type and timing of intervention. In GUCH, heart transplantations but also more recently in catheter-based valve interventions, the heart team’s opinion regarding the patient’s psychological condition and life and work implications are considered. Public expectations regarding risk control and the amount of shared information about the intervention have changed over the years. Developments on self-determination resulted in the opportunity for the patient, after informed consent, to make a decision which may deviate from the heart team’s advice.

In many heart centres the treatment plan is developed without the presence of the referring cardiologist and without meeting the patient in the outpatient clinic. The heart team process should be extended to enable shared decision-making. Patient preferences should be clearly documented and contribute to the decision. A few caveats for extended patient involvement have been mentioned, such as a possible patient preference for the least invasive approach [17] and the difficulty of participation by patients and families in a complex decision-making process [1]. In the future, the use of artificial intelligence for interpretation of data could possibly create improved interaction with patients for determining a sustainable and practical disease management plan.

## Patient safety and outcome measurement

Outcome measurement of the heart team process is indispensable to ensure adequate informed consent and patient safety. In the Netherlands, cardiology and cardiac surgery departments are united in a heart centre under joint supervision and with an allocated budget. This structure intends to combine the mutual interests and put patient care as primary focus for all the professionals involved. The heart centre provides the infrastructure, resources and embedding in the hospital system. Heart teams are the decision-making platforms including all the necessary expertise of medical and paramedical staff.

The outcomes of interventions and institutional performance have to be monitored. In the Netherlands, the Netherland Heart Registration (NHR) monitors outcomes of interventions in cardiovascular disease that matter most to patients. Annual reports confirm the high standards of care in the Netherlands and show only minor variations [20]. Outcomes of patients not receiving intervention and patient satisfaction are not yet monitored, resulting in an incomplete overview of the quality of decision-making in the heart team.

All the team member’s competences, training participation and professional behaviour should be monitored. Heart teams should receive feedback on decisions and outcomes including inter-institutional variance. To optimise this information, quality registries need to be extended with data used in the decision process (e.g. imaging data) and adopt advanced analysing techniques. Quality registries may use these patient data for quality control and improvement, even without patient informed consent [21].

Defining these actions under the responsibility of the heart team entails that the team’s leadership is well defined within the hospital and the heart centre hierarchy. This leadership includes responsibility for the organisation, quality of decision-making, monitoring the compliance to professional standards, but also the responsibility and accountability for outcomes—including appropriately dealing with adverse events. In heart centres, assignments of authority and responsibilities are not always clear.

## Heart team leadership and accountability

The complexity of cardiovascular care and the number of experts involved demand visible leadership. These challenges cannot be addressed by a case manager but should be continuously addressed within the heart centre and the heart team by the assigned leadership. Innovation in cardiovascular interventions involves highly complex patient care and the use of highest risk devices. The specialised heart teams gain their right to exist from the use of novel technology. Implant safety rightfully receives a lot of attention in the media because an appropriate monitoring and quality control system is lacking [22]. Current regulations explicitly prescribe instructions for use of products to be checked and decided upon by a specialised multidisciplinary heart team. Information about possible limited clinical experience or ongoing post-marketing follow-up studies should be shared with patients to make a risk-benefit trade-off. In addition, this implies that the heart team should monitor patients undergoing treatment with novel technology. National quality registries should play an important role in evaluating the performance of novel technologies in daily practice and providing this information to the heart teams. Secondly, it generates the obligation to report procedure- or device-related adverse events to stakeholders (e.g. manufacturer, heart centre or hospital management or healthcare authorities). As all heart team patients are followed, this should not create an extra workload but should result in increased awareness and willingness to report.

## Conclusion

The heart team concept is one of the most exciting and effective team modalities to ensure cost-effective application of invasive cardiac care. To deal with increased medical data that will be faced in the future, artificial intelligence could enable objective and effective interpretation of medical imaging and decision support. This technical support is indispensable to meet current patient and societal demands for informed consent, shared decision-making, outcome improvement and safety. Good heart team practice starts with motivated people. With the growing number of patients, procedures, medical data and participants, reliable planning and communication between the different team members of the heart team is indispensable. The heart team has great medical, ethical, legal, economic and societal responsibilities. The mission and focus of the heart team should be adjusted with clear leadership and accountability—similar to other high tech services in our society. In this way the heart team in the Netherlands will meet its responsibilities and be ready for the future.
